# Supplementary material for: New framework of Getis-Ord’s indexes associating spatial autocorrelation with interaction
Source: PLoS One. 2020 Jul 30;15(7):e0236765. doi: 10.1371/journal.pone.0236765 (PMC7392341; doi:10.1371/journal.pone.0236765)
Supplement: S1 File — It illustrates how to use new methods to calculate Getis-Ord’s indexes step by step using MS Excel. (PDF) [file pone.0236765.s003.pdf]

Simple approaches to calculating Getis-Ord's indexes using MS Excel

One of the six approaches to computing the global Getis-Ord's index and one of the three approaches to computing the local Getis-Ord's indexes are illustrated in this Supporting Information file. The capital cities of the 29 provinces, autonomous regions, and municipalities directly under the Central Government of China (CCC) are taken as an example. Before implementing a calculation, two datasets must be prepared as below: one is city population vector for 2010 year (Figure S1), and the other, spatial distance matrix (Figure S2).

	A	B
1	**City**	**Population size in 2010**
2	Beijing	15552378
3	Tianjin	8856234
4	Shijiazhuang	2756871
5	Taiyuan	3059130
6	Hohhot	1436617
7	Shenyang	5666061
8	Changchun	3289985
9	Harbin	4660837
10	Shanghai	17640842
11	Nanjing	5637120
12	Hangzhou	4411358
13	Hefei	3076276
14	Fuzhou	2675178
15	Nanchang	1917438
16	Jinan	3362744
17	Zhengzhou	3627841
18	Wuhan	7279628
19	Changsha	2876949
20	Guangzhou	9243138
21	Nanning	2424398
22	Chongqing	8671016
23	Chengdu	5893208
24	Guiyang	2430475
25	Kunming	3143524
26	Xi'an	4884116
27	Lanzhou	2381164
28	Xining	1141081
29	Yinchuan	1120306
30	Urumqi	2787993

Figure S1 The census data of the urban population of 29 Chinese cities in 2010

D	E	F	G	H	I	J	K	L	M	N	O	P
	Beijing	Tianjin	Shijiazhuang	Taiyuan	Hohhot	Shenyang	Changchun	Harbin	Shanghai	Nanjing	Hangzhou	Hefei
Beijing	0	137	277	508	667	741	1046	1288	1463	1160	1589	1074
Tianjin	137	0	419	650	804	728	1033	1275	1326	1023	1452	973
Shijiazhuang	277	419	0	231	871	1126	1431	1673	1267	964	1393	914
Taiyuan	508	650	231	0	640	1255	1560	1802	1498	1195	1624	1145
Hohhot	667	804	871	640	0	1408	1713	1955	2130	1827	2256	1777
Shenyang	741	728	1126	1255	1408	0	305	547	2054	1751	2180	1701
Changchun	1046	1033	1431	1560	1713	305	0	242	2359	2056	2485	2006
Harbin	1288	1275	1673	1802	1955	547	242	0	2601	2298	2727	2248
Shanghai	1463	1326	1267	1498	2130	2054	2359	2601	0	303	201	615
Nanjing	1160	1023	964	1195	1827	1751	2056	2298	303	0	429	312
Hangzhou	1589	1452	1393	1624	2256	2180	2485	2727	201	429	0	451
Hefei	1074	973	914	1145	1777	1701	2006	2248	615	312	451	0
Fuzhou	2334	2197	1915	2521	3303	2925	3230	3472	1180	1174	979	1196
Nanchang	1449	1444	1293	1544	2674	2151	2456	2698	844	838	643	478
Jinan	497	360	301	532	1164	1088	1393	1635	966	663	1092	613
Zhengzhou	689	831	412	577	1362	1538	1843	2085	998	695	1124	645
Wuhan	1225	1367	948	1179	1898	1972	2277	2519	1235	1231	1202	1181
Changsha	1587	1729	1310	1537	2256	2334	2639	2881	1207	1201	1006	1226
Guangzhou	2294	2436	2017	2243	2962	3041	3346	3588	1810	1804	1609	1829
Nanning	2566	2708	2289	2515	3234	3313	3618	3860	2082	2076	1881	2210
Chongqing	2087	2230	1810	1997	2637	2834	3139	3381	2516	2093	2315	2043
Chengdu	2042	2185	1765	1493	2133	2789	3094	3336	2351	2048	2552	1998
Guiyang	2539	2681	2262	2460	3100	3286	3591	3833	2053	2054	1852	2076
Kunming	3178	3320	2901	2593	3233	3925	4230	4472	2699	2693	2498	3098
Xi'an	1159	1301	923	651	1291	1906	2211	2453	1509	1206	1635	1156
Lanzhou	1811	1948	1599	1327	1144	2552	2857	3099	2185	1882	2311	1832
Xining	2092	2235	1815	1543	1360	2839	3144	3386	2401	2098	2527	2048
Yinchuan	1343	1480	1547	1316	676	2084	2389	2631	2355	2052	2481	2002
Urumqi	3768	3911	3491	3219	3036	4515	4820	5062	4077	3774	4065	3724

Figure S2 Railroad distance matrix of 29 Chinese cities by railway (partial results)

1 Calculating global Getis-Ord's index

Step 1: Unitizing size measures

First of all, I will show how to calculate global Getis-Ord's index using the three-step method through Microsoft Excel. The procedure of data unitization is as below. First, compute the total urban population of the 29 cities using the MS Excel function "sum". In cell B31, input a formula such as "=SUM(B2:B30)", press **Enter**, and it will yield a sum about 141903906. Second, unitize the population size. Select a region in the worksheet including cells C2-C30, input a formula such as "=B2:B30/B31", press **Ctrl** and **Shift** and **Enter** at the same time, thus yield the unitized array indicative of the size vector (**y**), which is shown in Figure S3.

	A	B	C	D	E
1	**City**	**Population size in 2010 (*x*)**	**Utilized variable (*y*)**	**Local Getis' G**	***y*G**
2	Beijing	15552378	0.109598	0.001831	0.000201
3	Tianjin	8856234	0.062410	0.002345	0.000146
4	Shijiazhuang	2756871	0.019428	0.002084	0.000040
5	Taiyuan	3059130	0.021558	0.001565	0.000034
6	Hohhot	1436617	0.010124	0.001106	0.000011
7	Shenyang	5666061	0.039929	0.001139	0.000045
8	Changchun	3289985	0.023185	0.001162	0.000027
9	Harbin	4660837	0.032845	0.000911	0.000030
10	Shanghai	17640842	0.124315	0.001278	0.000159
11	Nanjing	5637120	0.039725	0.001785	0.000071
12	Hangzhou	4411358	0.031087	0.001969	0.000061
13	Hefei	3076276	0.021679	0.001594	0.000035
14	Fuzhou	2675178	0.018852	0.000915	0.000017
15	Nanchang	1917438	0.013512	0.001490	0.000020
16	Jinan	3362744	0.023697	0.001751	0.000042
17	Zhengzhou	3627841	0.025565	0.001660	0.000042
18	Wuhan	7279628	0.051300	0.001277	0.000066
19	Changsha	2876949	0.020274	0.001346	0.000027
20	Guangzhou	9243138	0.065137	0.000776	0.000051
21	Nanning	2424398	0.017085	0.000798	0.000014
22	Chongqing	8671016	0.061105	0.000898	0.000055
23	Chengdu	5893208	0.041530	0.000938	0.000039
24	Guiyang	2430475	0.017128	0.001009	0.000017
25	Kunming	3143524	0.022152	0.000704	0.000016
26	Xi'an	4884116	0.034418	0.001204	0.000041
27	Lanzhou	2381164	0.016780	0.000934	0.000016
28	Xining	1141081	0.008041	0.000883	0.000007
29	Yinchuan	1120306	0.007895	0.000937	0.000007
30	Urumqi	2787993	0.019647	0.000420	0.000008
31	**Sum**	**141903906.00**	**1.000000**	**0.036710**	**0.001345**

Figure S3 The unitized data of the urban population of 29 Chinese cities in 2010 and the related calculation results

Step 2: Generating spatial weights matrix

The process of yielding a spatial weights matrix is as follows. First, convert the spatial distance matrix into a spatial contiguity matrix, **V**. Select a spatial weight function such as

$$v_{ij} = \begin{cases} r_{ij}^{-1}, & i \neq j \\ 0, & i = j \end{cases} . \tag{1}$$

In cell H33, input a formula such as "=IF(H2=0,0,1/H2)", press **Enter**, it will yield a number 0. Seize the bottom right corner of cell H33, drag it right and down, generate all the spatial contiguity values, which are shown in Figure S4. Second, summate the spatial contiguity values

using the following formula:

$$S = \sum_i \sum_j v_{ij} , \tag{2}$$

where S denotes an amount obtained as a of double summation. In cell AJ62, input a formula "=SUM(H33:AJ61)", press **Enter**, yield a sum around 0.6296. Third, transform the spatial contiguity matrix into a spatial weights matrix, **W**. In cell H64, input a formula "=H33/AJ62", press **Enter**, and it will yield the first value of spatial weights. Catch hold of the bottom right corner of cell H64, pull it right and down, and produce all the values of the spatial weights matrix, which are displayed in Figure S5.

	G	H	I	J	K	L	M	N	O	P	Q	R	S
32		Beijing	Tianjin	Shijiazhuang	Taiyuan	Hohhot	Shenyang	Changchun	Harbin	Shanghai	Nanjing	Hangzhou	Hefei
33	Beijing	0	0.0072993	0.0036101	0.0019685	0.0014993	0.0013495	0.000956	0.0007764	0.0006835	0.0008621	0.0006293	0.0009311
34	Tianjin	0.0072993	0	0.0023866	0.0015385	0.0012438	0.0013736	0.0009681	0.0007843	0.0007541	0.0009775	0.0006887	0.0010277
35	Shijiazhuang	0.0036101	0.0023866	0	0.004329	0.0011481	0.0008881	0.0006988	0.0005977	0.0007893	0.0010373	0.0007179	0.0010941
36	Taiyuan	0.0019685	0.0015385	0.004329	0	0.0015625	0.0007968	0.000641	0.0005549	0.0006676	0.0008368	0.0006158	0.0008734
37	Hohhot	0.0014993	0.0012438	0.0011481	0.0015625	0	0.0007102	0.0005838	0.0005115	0.0004695	0.0005473	0.0004433	0.0005627
38	Shenyang	0.0013495	0.0013736	0.0008881	0.0007968	0.0007102	0	0.0032787	0.0018282	0.0004869	0.0005711	0.0004587	0.0005879
39	Changchun	0.000956	0.0009681	0.0006988	0.000641	0.0005838	0.0032787	0	0.0041322	0.0004239	0.0004864	0.0004024	0.0004985
40	Harbin	0.0007764	0.0007843	0.0005977	0.0005549	0.0005115	0.0018282	0.0041322	0	0.0003845	0.0004352	0.0003667	0.0004448
41	Shanghai	0.0006835	0.0007541	0.0007893	0.0006676	0.0004695	0.0004869	0.0004239	0.0003845	0	0.0033003	0.0049751	0.001626
42	Nanjing	0.0008621	0.0009775	0.0010373	0.0008368	0.0005473	0.0005711	0.0004864	0.0004352	0.0033003	0	0.002331	0.0032051
43	Hangzhou	0.0006293	0.0006887	0.0007179	0.0006158	0.0004433	0.0004587	0.0004024	0.0003667	0.0049751	0.002331	0	0.0022173
44	Hefei	0.0009311	0.0010277	0.0010941	0.0008734	0.0005627	0.0005879	0.0004985	0.0004448	0.001626	0.0032051	0.0022173	0
45	Fuzhou	0.0004284	0.0004552	0.0005222	0.0003967	0.0003028	0.0003419	0.0003096	0.000288	0.0008475	0.0008518	0.0010215	0.0008361
46	Nanchang	0.0006901	0.0006925	0.0007734	0.0006477	0.000374	0.0004649	0.0004072	0.0003706	0.0011848	0.0011933	0.0015552	0.0020921
47	Jinan	0.0020121	0.0027778	0.0033223	0.0018797	0.0008591	0.0009191	0.0007179	0.0006116	0.0010352	0.0015083	0.0009158	0.0016313
48	Zhengzhou	0.0014514	0.0012034	0.0024272	0.0017331	0.0007342	0.0006502	0.0005426	0.0004796	0.001002	0.0014388	0.0008897	0.0015504
49	Wuhan	0.0008163	0.0007315	0.0010549	0.0008482	0.0005269	0.0005071	0.0004392	0.000397	0.0008097	0.0008123	0.0008319	0.0008467
50	Changsha	0.0006301	0.0005784	0.0007634	0.0006506	0.0004433	0.0004284	0.0003789	0.0003471	0.0008285	0.0008326	0.000994	0.0008157
51	Guangzhou	0.0004359	0.0004105	0.0004958	0.0004458	0.0003376	0.0003288	0.0002989	0.0002787	0.0005525	0.0005543	0.0006215	0.0005467
52	Nanning	0.0003897	0.0003693	0.0004369	0.0003976	0.0003092	0.0003018	0.0002764	0.0002591	0.0004803	0.0004817	0.0005316	0.0004525
53	Chongqing	0.0004792	0.0004484	0.0005525	0.0005008	0.0003792	0.0003529	0.0003186	0.0002958	0.0003975	0.0004778	0.000432	0.0004895
54	Chengdu	0.0004897	0.0004577	0.0005666	0.0006698	0.0004688	0.0003586	0.0003232	0.0002998	0.0004254	0.0004883	0.0003918	0.0005005
55	Guiyang	0.0003939	0.000373	0.0004421	0.0004065	0.0003226	0.0003043	0.0002785	0.0002609	0.0004871	0.0004869	0.00054	0.0004817
56	Kunming	0.0003147	0.0003012	0.0003447	0.0003857	0.0003093	0.0002548	0.0002364	0.0002236	0.0003705	0.0003713	0.0004003	0.0003228
57	Xi'an	0.0008628	0.0007686	0.0010834	0.0015361	0.0007746	0.0005247	0.0004523	0.0004077	0.0006627	0.0008292	0.0006116	0.0008651
58	Lanzhou	0.0005522	0.0005133	0.0006254	0.0007536	0.0008741	0.0003918	0.00035	0.0003227	0.0004577	0.0005313	0.0004327	0.0005459
59	Xining	0.000478	0.0004474	0.000551	0.0006481	0.0007353	0.0003522	0.0003181	0.0002953	0.0004165	0.0004766	0.0003957	0.0004883
60	Yinchuan	0.0007446	0.0006757	0.0006464	0.0007599	0.0014793	0.0004798	0.0004186	0.0003801	0.0004246	0.0004873	0.0004031	0.0004995
61	Urumqi	0.0002654	0.0002557	0.0002865	0.0003107	0.0003294	0.0002215	0.0002075	0.0001976	0.0002453	0.000265	0.000246	0.0002685

Figure S4 Spatial contiguity matrix of 29 Chinese cities in 2010 (partial results)

Step 3: Computing global Getis-Ord's index

By means of the formula $G=\mathbf{y}^T\mathbf{W}\mathbf{y}$, it is easy to calculate global Getis-Ord's index (G) using Excel functions "mmult" and "transpose". According to the number and result arrangement in the same worksheet, in any cell, say, B34, you can input a formula as below:

"=MMULT(MMULT(TRANSPOSE(C2:C30),H64:AJ92),C2:C30)"

Pressing **Ctrl** and **Shift** and **Enter** at the same time yields the global Getis-Ord's index value immediately. The result is about G=0.001345, and the details for calculations can be found in File S2.

	G	H	I	J	K	L	M	N	O	P	Q	R	S
63		Beijing	Tianjin	Shijiazhuang	Taiyuan	Hohhot	Shenyang	Changchun	Harbin	Shanghai	Nanjing	Hangzhou	Hefei
64	Beijing	0	0.0115931	0.0057338	0.0031265	0.0023812	0.0021434	0.0015184	0.0012331	0.0010856	0.0013692	0.0009995	0.0014788
65	Tianjin	0.0115931	0	0.0037906	0.0024435	0.0019754	0.0021817	0.0015375	0.0012457	0.0011978	0.0015525	0.0010938	0.0016323
66	Shijiazhuang	0.0057338	0.0037906	0	0.0068755	0.0018235	0.0014105	0.0011099	0.0009493	0.0012536	0.0016476	0.0011402	0.0017377
67	Taiyuan	0.0031265	0.0024435	0.0068755	0	0.0024816	0.0012655	0.0010181	0.0008814	0.0010602	0.0013291	0.000978	0.0013871
68	Hohhot	0.0023812	0.0019754	0.0018235	0.0024816	0	0.001128	0.0009272	0.0008124	0.0007457	0.0008693	0.000704	0.0008938
69	Shenyang	0.0021434	0.0021817	0.0014105	0.0012655	0.001128	0	0.0052074	0.0029036	0.0007732	0.0009071	0.0007286	0.0009337
70	Changchun	0.0015184	0.0015375	0.0011099	0.0010181	0.0009272	0.0052074	0	0.006563	0.0006733	0.0007725	0.0006391	0.0007917
71	Harbin	0.0012331	0.0012457	0.0009493	0.0008814	0.0008124	0.0029036	0.006563	0	0.0006106	0.0006911	0.0005824	0.0007065
72	Shanghai	0.0010856	0.0011978	0.0012536	0.0010602	0.0007457	0.0007732	0.0006733	0.0006106	0	0.0052417	0.0079017	0.0025825
73	Nanjing	0.0013692	0.0015525	0.0016476	0.0013291	0.0008693	0.0009071	0.0007725	0.0006911	0.0052417	0	0.0037022	0.0050905
74	Hangzhou	0.0009995	0.0010938	0.0011402	0.000978	0.000704	0.0007286	0.0006391	0.0005824	0.0079017	0.0037022	0	0.0035216
75	Hefei	0.0014788	0.0016323	0.0017377	0.0013871	0.0008938	0.0009337	0.0007917	0.0007065	0.0025825	0.0050905	0.0035216	0
76	Fuzhou	0.0006805	0.0007229	0.0008294	0.00063	0.0004809	0.000543	0.0004917	0.0004574	0.001346	0.0013529	0.0016223	0.001328
77	Nanchang	0.0010961	0.0010999	0.0012283	0.0010287	0.000594	0.0007384	0.0006467	0.0005887	0.0018818	0.0018953	0.0024701	0.0033227
78	Jinan	0.0031957	0.0044118	0.0052766	0.0029854	0.0013645	0.0014598	0.0011402	0.0009714	0.0016442	0.0023955	0.0014544	0.0025909
79	Zhengzhou	0.0023052	0.0019113	0.003855	0.0027526	0.0011661	0.0010327	0.0008618	0.0007618	0.0015914	0.0022853	0.001413	0.0024624
80	Wuhan	0.0012965	0.0011619	0.0016754	0.0013471	0.0008368	0.0008054	0.0006975	0.0006305	0.001286	0.0012902	0.0013213	0.0013448
81	Changsha	0.0010008	0.0009186	0.0012124	0.0010333	0.000704	0.0006805	0.0006018	0.0005513	0.0013159	0.0013224	0.0015788	0.0012955
82	Guangzhou	0.0006923	0.000652	0.0007874	0.0007081	0.0005362	0.0005223	0.0004747	0.0004427	0.0008775	0.0008804	0.0009871	0.0008684
83	Nanning	0.000619	0.0005865	0.0006939	0.0006315	0.0004911	0.0004794	0.000439	0.0004115	0.0007628	0.0007651	0.0008444	0.0007187
84	Chongqing	0.000761	0.0007122	0.0008775	0.0007953	0.0006023	0.0005604	0.000506	0.0004698	0.0006313	0.0007588	0.0006861	0.0007774
85	Chengdu	0.0007778	0.0007269	0.0008999	0.0010638	0.0007446	0.0005695	0.0005133	0.0004761	0.0006756	0.0007755	0.0006224	0.0007949
86	Guiyang	0.0006255	0.0005924	0.0007021	0.0006456	0.0005123	0.0004833	0.0004423	0.0004144	0.0007736	0.0007732	0.0008576	0.0007651
87	Kunming	0.0004998	0.0004784	0.0005475	0.0006125	0.0004913	0.0004046	0.0003755	0.0003552	0.0005885	0.0005898	0.0006358	0.0005127
88	Xi'an	0.0013704	0.0012208	0.0017207	0.0024397	0.0012302	0.0008333	0.0007183	0.0006475	0.0010525	0.001317	0.0009714	0.0013739
89	Lanzhou	0.000877	0.0008153	0.0009933	0.0011969	0.0013883	0.0006224	0.0005559	0.0005125	0.0007269	0.0008439	0.0006873	0.0008669
90	Xining	0.0007592	0.0007106	0.0008751	0.0010293	0.0011678	0.0005594	0.0005052	0.0004691	0.0006615	0.000757	0.0006285	0.0007755
91	Yinchuan	0.0011826	0.0010731	0.0010267	0.0012069	0.0023495	0.0007621	0.0006648	0.0006037	0.0006744	0.000774	0.0006402	0.0007933
92	Urumqi	0.0004215	0.0004061	0.000455	0.0004934	0.0005231	0.0003518	0.0003295	0.0003138	0.0003896	0.0004208	0.0003907	0.0004265

Figure S5 Spatial weights matrix of 29 Chinese cities in 2010 (partial results)

2 Calculating local Getis-Ord's indexes and mutual energy indexes

Step 1: Computing local Getis-Ord's *G* and potential energy indexes

Next, I will show how to calculate local Getis-Ord's indexes and the related energy indexes by means of Microsoft Excel. Using matrix multiplication, we can compute the local Getis-Ord's indexes easily by means of Excel. Select a region in the worksheet including cells D2-D30, input a formula based on multiplication of matrices, that is, "=MMULT(H64:AJ92,C2:C30)"; pressing **Ctrl** and **Shift** and **Enter** at the same time yields the vector for the local Getis-Ord's indexes (**G**),

which has been displayed in Figure S3. The local Getis-Ord's indexes are equal to the corresponding potential energy indexes.

Step 2: Computing mutual energy indexes

Using array multiplication, we can compute the mutual energy indexes. Please note that the array multiplication differs from the matrix multiplication in Excel. Select a region in the worksheet including cells E2-E30, input a formula based on multiplication of arrays such as "=C2:C30*D2:D30"; pressing **Ctrl** and **Shift** and **Enter** at the same time yields the vector for the mutual energy indexes (**E**), which has been displayed in Figure S3. The sum of the mutual energy indexes is equal to the global Getis-Ord's index, G=0.001345.

3 Drawing Getis-Ord's scatterplots

Finally, I will show how to draw a Getis-Ord's scatterplot in Microsoft Excel. By means of matrix multiplication, we can compute the two variables, $f=\mathbf{z}^{T}\mathbf{z}\mathbf{W}\mathbf{z}$ and $f^{*}=\mathbf{z}\mathbf{z}^{T}\mathbf{W}\mathbf{z}$. Select a region in the worksheet including cells D2-D30, input a formula based on multiplication of matrices, "=MMULT(TRANSPOSE(C2:C30),C2:C30)*MMULT(H64:AJ92,C2:C30)"; pressing **Ctrl** and **Shift** and **Enter** at the same time yields the vector for the variable f; then, select a region in the worksheet including cells E2-E30, input a formula based on matrix multiplication, "=MMULT(MMULT(C2:C30,TRANSPOSE(C2:C30)),MMULT(H64:AJ92,C2:C30))"; pressing **Ctrl** and **Shift** and **Enter** at the same time yields the vector for the variable f^{*} (Figure S6). Using the unitized vector y as x-axis, and using the variables f and f^{*} as a y-axis, we can make a Getis-Ord scatterplot (Figure S7).

	A	B	C	D	E
1	**City**	**Population size in 2010 (x)**	**Utilized variable (y)**	f	f^*
2	Beijing	15552378	0.109598	0.000103	0.000147
3	Tianjin	8856234	0.062410	0.000132	0.000084
4	Shijiazhuang	2756871	0.019428	0.000117	0.000026
5	Taiyuan	3059130	0.021558	0.000088	0.000029
6	Hohhot	1436617	0.010124	0.000062	0.000014
7	Shenyang	5666061	0.039929	0.000064	0.000054
8	Changchun	3289985	0.023185	0.000065	0.000031
9	Harbin	4660837	0.032845	0.000051	0.000044
10	Shanghai	17640842	0.124315	0.000072	0.000167
11	Nanjing	5637120	0.039725	0.000100	0.000053
12	Hangzhou	4411358	0.031087	0.000110	0.000042
13	Hefei	3076276	0.021679	0.000089	0.000029
14	Fuzhou	2675178	0.018852	0.000051	0.000025
15	Nanchang	1917438	0.013512	0.000084	0.000018
16	Jinan	3362744	0.023697	0.000098	0.000032
17	Zhengzhou	3627841	0.025565	0.000093	0.000034
18	Wuhan	7279628	0.051300	0.000072	0.000069
19	Changsha	2876949	0.020274	0.000076	0.000027
20	Guangzhou	9243138	0.065137	0.000044	0.000088
21	Nanning	2424398	0.017085	0.000045	0.000023
22	Chongqing	8671016	0.061105	0.000050	0.000082
23	Chengdu	5893208	0.041530	0.000053	0.000056
24	Guiyang	2430475	0.017128	0.000057	0.000023
25	Kunming	3143524	0.022152	0.000040	0.000030
26	Xi'an	4884116	0.034418	0.000068	0.000046
27	Lanzhou	2381164	0.016780	0.000052	0.000023
28	Xining	1141081	0.008041	0.000050	0.000011
29	Yinchuan	1120306	0.007895	0.000053	0.000011
30	Urumqi	2787993	0.019647	0.000024	0.000026

Figure S6 The variables for the Getis-Ord's scatterplot of the 29 Chinese cities in 2010 (based on power-law distance decay

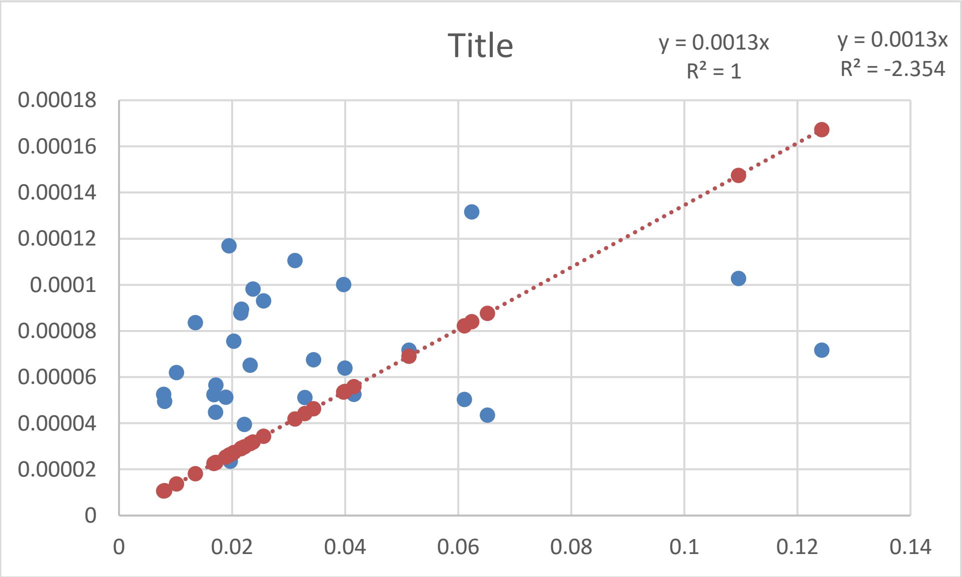

Figure S7 A Getis-Ord's scatterplot for the 29 Chinese cities in 2010)
